# Unusual thermolysis of aza­cyclic allene under microwave conditions: crystal structure of (3*RS*,3a*SR*,8*RS*,8a*RS*)-methyl 5,6-dimeth­oxy-3a,10-dimethyl-1-phenyl-3,3a,8,8a-tetra­hydro-3,8-(epimino­methano)­cyclo­penta­[*a*]indene-2-carboxyl­ate from synchrotron X-ray diffraction

**DOI:** 10.1107/S2056989017014864

**Published:** 2017-10-24

**Authors:** Le Tuan Anh, Alexander A. Titov, Maxim S. Kobzev, Leonid G. Voskressensky, Alexey V. Varlamov, Pavel V. Dorovatovskii, Victor N. Khrustalev

**Affiliations:** a Department of Pharmaceutical Chemistry, Faculty of Chemistry, VNU University of Science, 19 Le Thanh Tong, Hoan Kiem, Hanoi 100000, Vietnam; b Organic Chemistry Department, Peoples’ Friendship University of Russia, (RUDN University), Miklukho-Maklay St., 6, Moscow 117198, Russian Federation; c National Research Centre "Kurchatov Institute", 1 Acad. Kurchatov Sq., Moscow 123182, Russian Federation; d Inorganic Chemistry Department, Peoples’ Friendship University of Russia, (RUDN University), Miklukho-Maklay St., 6, Moscow 117198, Russian Federation

**Keywords:** crystal structure, aza­cyclic allenes, thermolysis, microwave synthesis, (epimino­methano)­cyclo­penta­[*a*]indene, 3-benzazepine, synchrotron X-ray diffraction

## Abstract

The title compound, C_25_H_27_NO_4_ (I), the product of the unusual thermolysis of aza­cyclic allene methyl 10,11-dimeth­oxy-3,8-dimethyl-6-phenyl-3-aza­benzo[*d*]cyclo­deca-4,6,7-triene-5-carboxyl­ate, represents a bicyclic heterosystem and crystallizes in the monoclinic space group *P*2_1_/*c* with three crystallographically independent mol­ecules in the unit cell. These independent mol­ecules adopt very similar geometries and differ only in the conformations of the two meth­oxy substituents on the benzene ring. In two of the three independent mol­ecules, both meth­oxy groups are almost coplanar with the benzene ring [the C—C—O—Me torsion angles are 10.8 (2), 12.3 (2), 9.1 (2) and 13.6 (3)°], whereas in the third mol­ecule, one of the meth­oxy groups is practically coplanar to and the other meth­oxy group is roughly perpendicular to the benzene ring, the C—C—O—Me torsion angles being 14.1 (2) and 76.5 (2)°. The mol­ecule of (I) comprises a fused tetra­cyclic system containing two five-membered rings (cyclo­pentenes) and two six-membered rings (piperidine and benzene). The five-membered rings have the usual envelope conformation, with the methyl-subsituted C atom as the flap in each molecule, and the six-membered piperidine ring has a chair conformation. The methyl substituent at the N atom occupies the sterically favourable equatorial position. The carboxyl­ate group lies almost within the basal plane of the parent cyclo­pentene ring [making dihedral angle of 11.68 (8), 18.94 (9) and 15.16 (9)° in the three independent mol­ecules], while the phenyl substituent is twisted by 48.26 (6), 42.04 (6) and 41.28 (6)° (for the three independent mol­ecules) relative to this plane. In the crystal, mol­ecules of (I) form stacks along the *b*-axis direction. The mol­ecules are arranged at van der Waals distances.

## Abstract 2

The structure of methyl 5,6-dimeth­oxy-3a,10-dimethyl-1-phenyl-3,3a,8,8a-tetra­hydro-3,8-(epimino­methano)­cyclo­penta­[*a*]indene-2-carboxyl­ate, the product of unusual thermolysis of aza­cyclic allene under microvawe conditions, was studied by synchrotron X-ray diffraction.

## Chemical context   

The allene fragment is a part of some natural compounds – steroids, prostaglandins, amines acids, nucleosides. The vast majority of them contain a non-cyclic allene fragment. Ten-membered steroids with an allene fragment in bacteria have already been studied (Batzold & Robinson, 1975 ▸, 1976 ▸; Batzold *et al.*, 1977 ▸). Bicyclic lactones comprising ten-membered sesquiterpene allenes have been isolated from the *Vernonia* plants of South America (Bohlmann *et al.*, 1980 ▸; Jakupovic *et al.*, 1985 ▸; Warning *et al.*, 1987 ▸). Natural functionalized allenes are inhibitors of enzymes that exhibit cytotoxic and anti­viral activity (Krause & Hoffmann-Röder, 2004 ▸). However, heterocyclic allene systems are practically unexplored. Nitro­gen-containing nona­trienes-3,6,7 were obtained for the first time in 1984 with a yield of 8–10% (Sashida & Tsuchiya, 1984 ▸). Further, the synthesis of 1-aza­deca­(undeca­)-4,5-dien-2-ones has been carried out (Perscheid *et al.*, 2011 ▸).

Recently, we proposed a relatively simple synthesis of benzo­aza­deca­trienes-4,6,7 from 1*R*-1-phenyl­ethynyl tetra­hydro­iso­quinolines and activated terminal alkynes in tri­fluoro­ethanol at 256 K (Voskressensky *et al.*, 2017 ▸). However, the thermal transformations of such strained systems have not yet been studied.
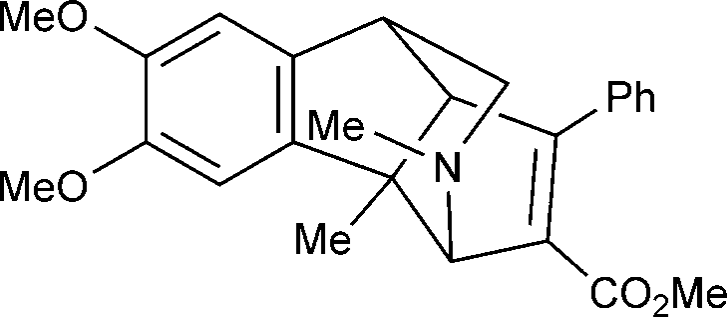


In this work, the thermolysis of an aza­cyclic allene under microwave conditions was carried out by our research group. The structure of the product of this unusual reaction was established unambiguously by synchrotron X-ray diffraction study.

## Structural commentary   

**Figure 1:**
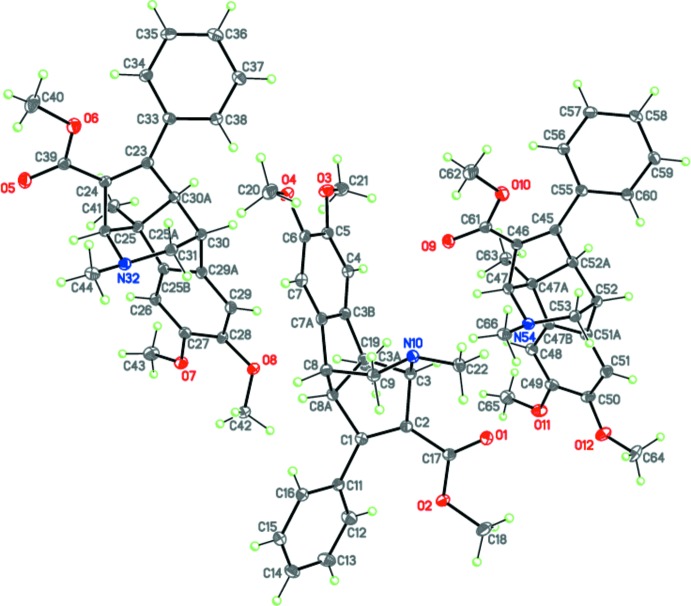
The mol­ecular structure of (I) showing the three crystallographically independent mol­ecules. Displacement ellipsoids are shown at the 50% probability level. H atoms are shown as small spheres of arbitrary radius.

The title compound crystallizes with three crystallographically independent mol­ecules (*A*, *B* and *C*, containing atoms N10, N32 and N54, respectively) in the unit cell (Fig. 1 ▸). These independent mol­ecules adopt very similar geometries and differ only in the conformations of the two meth­oxy substituents at the benzene ring. In two of the three independent mol­ecules, both meth­oxy groups are almost coplanar to the benzene ring [the C—C—O—Me torsion angles are 10.8 (2) and 12.3 (2)° in mol­ecule *A* and 9.1 (2) and 13.6 (3)° in *B*], whereas in the third mol­ecule, *C*, one of the meth­oxy groups is practically coplanar with and the other meth­oxy group is roughly perpendicular to the benzene ring, the C—C—O—Me torsion angles being 14.1 (2) and 76.5 (2)°].

The mol­ecule of (I) comprises a fused tetra­cyclic system containing two five-membered rings (cyclo­pentenes) and two six-membered rings (piperidine and benzene) (Fig. 1 ▸). The five-membered rings have the usual envelope conformation, with the methyl-subsituted C atom as the flap in each molecule, and the six-membered piperidine ring has a chair conformation. The methyl substituent at the nitro­gen atom occupies the sterically favourable equatorial position. The carboxyl­ate group lies almost within the basal plane of the parent cyclo­pentene ring, making dihedral angles of 11.68 (8), 18.94 (9) and 15.16 (9)°, respectively, in mol­ecules *A*, *B* and *C*, while the phenyl substituent is twisted by 48.26 (6), 42.04 (6) and 41.28 (6)° relative to this plane in mol­ecules *A*, *B* and *C*, respectively.

The title mol­ecule possesses four asymmetric centers at the C3, C3*A*, C8 and C8*A* carbon atoms and can have potentially numerous diastereomers. The crystal of (I) is racemic and consists of enanti­omeric pairs with the following relative configuration of the centers: *rac*-3R*,3aS*,8R*,8aR*.

## Supra­molecular features   

**Figure 2:**
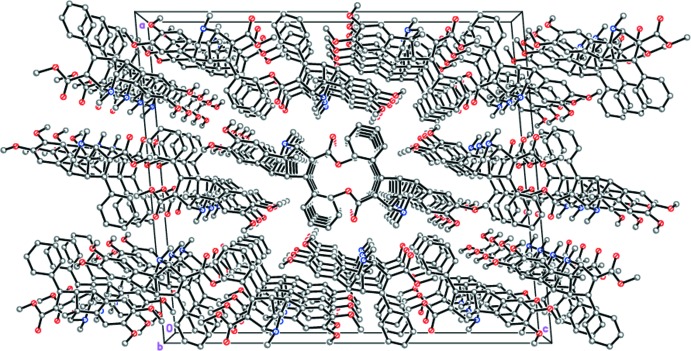
Crystal structure of (I) illustrating the stacks along [010]. For clarity, H atoms have been omitted.

The crystal packing motif of mol­ecules of (I) is stacking along the crystallographic *b* axis (Fig. 2 ▸). The mol­ecules are arranged at van der Waals distances.

## Synthesis and crystallization   

**Figure 3:**
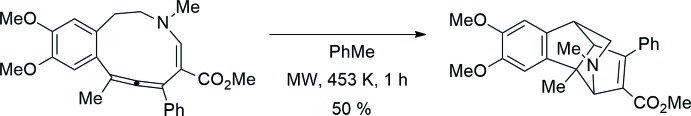
Thermolysis of aza­cyclic allene methyl 10,11-dimeth­oxy-3,8-dimethyl-6-phenyl-3-aza­benzo[*d*]cyclo­deca-4,6,7-triene-5-carboxyl­ate under microwave conditions.

A stirred solution of cyclic allene (0.13 g, 0.32 mmol) in toluene (5 mL) was heated at 453 K for 1 h in a microwave reactor (Anton Paar Monowave 300) (Fig. 3 ▸). The solvent was evaporated *in vacuo*, and the residue recrystallized from ether to give 60 mg of colourless crystals of (I) in a yield of 50%, m.p. = 422–424 K (ether).

^1^H NMR (CDCl_3_, 600 MHz): *δ* = 1.47 (3H, *s*, 3a-Me), 2.21 (3H, *s*, NMe), 2.47 (1H, *dd*, *J* = 2.5 Hz, *J* = 10.7 Hz, 9-CH), 2.65 (1H, *dd*, *J* = 2.5 Hz, *J* = 10.7 Hz, 9-CH), 3.09–3.10 (1H, *m*, 8-CH), 3.22 (1H, *d*, *J* = 5.0 Hz, 8a-CH), 3.67 (1H, *d*, *J* = 1.7 Hz, 3-CH), 3.69 (3H, *s*, OMe), 3.88 (3H, *s*, OMe), 3.91 (3H, *s*, OMe), 6.73 (1H, *s*, H7), 6.74 (1H, *s*, H4), 7.35–7.40 (3H, *m*, Ph), 7.47–7.48 (2*H*, *m*, Ph); ^13^C NMR (DMSO-*d_6_*, 150 MHz): *δ* = 18.3, 42.3, 42.6, 51.1, 51.2, 55.7, 55.8, 59.6, 67.9, 72.8, 106.9, 107.1, 124.9, 127.9 (2 C), 128.0 (2 C), 128.5, 135.1, 137.1, 137.3, 147.8, 148.3, 151.1, 166.3; *m*/*z*: 406 [*M*+H]^+^. Analysis calculated for C_25_H_27_NO_4_ (%): C 74.03, H 6.71, N 3.45; found (%): C 74.04, H 6.71, N 3.41.

## Refinement   

Crystal data, data collection and structure refinement details are summarized in Table 1 ▸. The X-ray diffraction study was carried out on the ‘Belok’ beamline of the National Research Center ‘Kurchatov Institute’ (Moscow, Russian Federation) using a Rayonix SX165 CCD detector. A total of 720 images were collected using an oscillation range of 1.0° (φ scan mode, two different crystal orientations) and corrected for absorption using the *Scala* program (Evans, 2006 ▸). The data were indexed, integrated and scaled using the utility *i*MOSFLM in *CCP4* (Battye *et al.*, 2011 ▸).

Hydrogen atoms were placed in calculated positions with C—H = 0.95–1.00 Å and refined in the riding model with fixed isotropic displacement parameters [*U*_iso_(H) = 1.5*U*_eq_(C) for the CH_3_-groups and 1.2*U*_eq_(C) for the other groups].

A rather large number of reflections have been omitted for the following reasons: (i) In order to achieve better *I*σ statistics for high-angle reflections, we selected exposure times so as to admit a minor fraction of intensity overloads in the low-angle part of the detector. These low-angle reflections with imprecisely measured intensities were excluded from the final steps of the refinement. (ii) In the present setup of the synchrotron diffractometer, the low-temperature device eclipses a small region of the 2D detector near the high-angle limit. This small shadowed region was not masked during integration of the diffraction frames, which erroneously resulted in zero intensity for some reflections.

## Supplementary Material

Crystal structure: contains datablock(s) global, I. DOI: 10.1107/S2056989017014864/yk2110sup1.cif

Structure factors: contains datablock(s) I. DOI: 10.1107/S2056989017014864/yk2110Isup2.hkl

CCDC reference: 1579823

Additional supporting information:  crystallographic information; 3D view; checkCIF report

## Figures and Tables

**Table 1:** Experimental details

Crystal data	
Chemical formula	C_25_H_27_NO_4_
*M*_r_	405.47
Crystal system, space group	Monoclinic, *P*2_1_/*c*
Temperature (K)	100
*a*, *b*, *c* (Å)	26.404 (5), 7.9303 (16), 30.720 (6)
β (°)	95.09 (3)
*V* (Å^3^)	6407 (2)
*Z*	12
Radiation type	Synchrotron, λ = 0.96990 Å
μ (mm^−1^)	0.18
Crystal size (mm)	0.25 × 0.24 × 0.21

Data collection	
Diffractometer	Rayonix SX165 CCD
Absorption correction	Multi-scan (*SCALA*; Evans, 2006 ▸)
*T*_min_, *T*_max_	0.940, 0.951
No. of measured, independent and observed [*I* > 2σ(*I*)] reflections	74044, 13622, 10086
*R*_int_	0.071
(sin θ/λ)_max_ (Å^−1^)	0.659

Refinement	
*R*[*F* ^2^ > 2σ(*F* ^2^)], *wR*(*F* ^2^), *S*	0.053, 0.146, 1.07
No. of reflections	13622
No. of parameters	827
H-atom treatment	H-atom parameters constrained
Δρ_max_, Δρ_min_ (e Å^−3^)	0.46, −0.38

Computer programs: *Marccd* (Doyle, 2011 ▸), *i* *MOSFLM* (Battye *et al.*, 2011 ▸), *SHELXT* (Sheldrick, 2015*a* ▸), *SHELXL2014* (Sheldrick, 2015*b* ▸) and *SHELXTL* (Sheldrick, 2008 ▸).

## supplementary crystallographic information

### Crystal data

**Table:**

C_25_H_27_NO_4_	*F*(000) = 2592
*M**_r_* = 405.47	*D*_x_ = 1.261 Mg m^−^^3^
Monoclinic, *P*2_1_/*c*	Synchrotron radiation, λ = 0.96990 Å
*a* = 26.404 (5) Å	Cell parameters from 600 reflections
*b* = 7.9303 (16) Å	θ = 3.6–36.0°
*c* = 30.720 (6) Å	µ = 0.18 mm^−^^1^
β = 95.09 (3)°	*T* = 100 K
*V* = 6407 (2) Å^3^	Prism, colourless
*Z* = 12	0.25 × 0.24 × 0.21 mm

### Data collection

**Table:**

Rayonix SX165 CCD diffractometer	10086 reflections with *I* > 2σ(*I*)
/f scan	*R*_int_ = 0.071
Absorption correction: multi-scan (*Scala*; Evans, 2006)	θ_max_ = 39.7°, θ_min_ = 3.5°
*T*_min_ = 0.940, *T*_max_ = 0.951	*h* = −33→33
74044 measured reflections	*k* = −8→10
13622 independent reflections	*l* = −39→39

### Refinement

**Table:**

Refinement on *F*^2^	Secondary atom site location: difference Fourier map
Least-squares matrix: full	Hydrogen site location: inferred from neighbouring sites
*R*[*F*^2^ > 2σ(*F*^2^)] = 0.053	H-atom parameters constrained
*wR*(*F*^2^) = 0.146	*w* = 1/[σ^2^(*F*_o_^2^) + (0.0696*P*)^2^ + 0.8571*P*] where *P* = (*F*_o_^2^ + 2*F*_c_^2^)/3
*S* = 1.07	(Δ/σ)_max_ = 0.001
13622 reflections	Δρ_max_ = 0.46 e Å^−^^3^
827 parameters	Δρ_min_ = −0.38 e Å^−^^3^
0 restraints	Extinction correction: SHELXL2014 (Sheldrick, 2015b), Fc^*^=kFc[1+0.001xFc^2^λ^3^/sin(2θ)]^-1/4^
Primary atom site location: difference Fourier map	Extinction coefficient: 0.00120 (18)

### Special details

**Table:**

Geometry. All esds (except the esd in the dihedral angle between two l.s. planes) are estimated using the full covariance matrix. The cell esds are taken into account individually in the estimation of esds in distances, angles and torsion angles; correlations between esds in cell parameters are only used when they are defined by crystal symmetry. An approximate (isotropic) treatment of cell esds is used for estimating esds involving l.s. planes.

### Fractional atomic coordinates and isotropic or equivalent isotropic displacement parameters (Å^2^)

**Table:**

	*x*	*y*	*z*	*U*_iso_*/*U*_eq_	
O1	0.27574 (4)	0.67806 (16)	0.64919 (4)	0.0291 (3)	
O2	0.19523 (4)	0.75446 (16)	0.65782 (4)	0.0293 (3)	
O3	0.30254 (4)	0.29541 (15)	0.38931 (4)	0.0266 (3)	
O4	0.26028 (4)	0.55159 (15)	0.35018 (3)	0.0244 (3)	
C1	0.16537 (6)	0.6161 (2)	0.57029 (5)	0.0201 (3)	
C2	0.21299 (6)	0.6304 (2)	0.59036 (5)	0.0198 (3)	
C3	0.25165 (6)	0.5938 (2)	0.55724 (5)	0.0195 (3)	
H3	0.2835	0.5432	0.5717	0.023*	
C3A	0.22152 (6)	0.4638 (2)	0.52660 (5)	0.0195 (3)	
C3B	0.23500 (6)	0.4674 (2)	0.47966 (5)	0.0197 (3)	
C4	0.26706 (6)	0.3619 (2)	0.45845 (5)	0.0210 (4)	
H4	0.2842	0.2711	0.4736	0.025*	
C5	0.27349 (6)	0.3925 (2)	0.41453 (5)	0.0210 (4)	
C6	0.24940 (6)	0.5322 (2)	0.39281 (5)	0.0206 (4)	
C7	0.21706 (6)	0.6363 (2)	0.41435 (5)	0.0218 (4)	
H7	0.2006	0.7297	0.3998	0.026*	
C7A	0.20952 (6)	0.6002 (2)	0.45771 (5)	0.0196 (3)	
C8	0.17978 (6)	0.6978 (2)	0.48975 (5)	0.0205 (4)	
H8	0.1478	0.7487	0.4756	0.025*	
C8A	0.16948 (6)	0.5569 (2)	0.52346 (5)	0.0201 (3)	
H8A	0.1407	0.4817	0.5127	0.024*	
C9	0.21579 (6)	0.8298 (2)	0.51258 (5)	0.0207 (3)	
H9A	0.2247	0.9154	0.4911	0.025*	
H9B	0.1985	0.8878	0.5357	0.025*	
N10	0.26251 (5)	0.74768 (17)	0.53217 (4)	0.0200 (3)	
C11	0.11459 (6)	0.6438 (2)	0.58631 (5)	0.0205 (3)	
C12	0.10117 (7)	0.5756 (2)	0.62547 (6)	0.0291 (4)	
H12	0.1255	0.5114	0.6431	0.035*	
C13	0.05300 (7)	0.5997 (2)	0.63919 (6)	0.0330 (4)	
H13	0.0445	0.5506	0.6658	0.040*	
C14	0.01719 (6)	0.6951 (2)	0.61416 (6)	0.0294 (4)	
H14	−0.0156	0.7138	0.6237	0.035*	
C15	0.03000 (6)	0.7628 (2)	0.57498 (6)	0.0286 (4)	
H15	0.0058	0.8286	0.5577	0.034*	
C16	0.07783 (6)	0.7357 (2)	0.56054 (5)	0.0248 (4)	
H16	0.0856	0.7797	0.5331	0.030*	
C17	0.23166 (6)	0.6887 (2)	0.63484 (5)	0.0216 (4)	
C18	0.21198 (7)	0.8065 (3)	0.70192 (5)	0.0349 (5)	
H18A	0.2401	0.8868	0.7012	0.052*	
H18B	0.2235	0.7076	0.7192	0.052*	
H18C	0.1837	0.8602	0.7152	0.052*	
C19	0.22182 (6)	0.2883 (2)	0.54690 (5)	0.0243 (4)	
H19A	0.1990	0.2142	0.5287	0.036*	
H19B	0.2103	0.2954	0.5763	0.036*	
H19C	0.2564	0.2424	0.5487	0.036*	
C20	0.24521 (7)	0.7058 (2)	0.32898 (5)	0.0300 (4)	
H20A	0.2585	0.7100	0.3002	0.045*	
H20B	0.2588	0.8010	0.3467	0.045*	
H20C	0.2080	0.7124	0.3254	0.045*	
C21	0.32021 (7)	0.1375 (2)	0.40771 (6)	0.0308 (4)	
H21A	0.3390	0.0767	0.3865	0.046*	
H21B	0.2911	0.0697	0.4149	0.046*	
H21C	0.3427	0.1583	0.4343	0.046*	
C22	0.29463 (6)	0.8685 (2)	0.55748 (5)	0.0239 (4)	
H22A	0.2753	0.9199	0.5798	0.036*	
H22B	0.3057	0.9564	0.5380	0.036*	
H22C	0.3245	0.8103	0.5716	0.036*	
O5	0.04526 (4)	0.27952 (16)	0.18932 (4)	0.0306 (3)	
O6	0.12346 (4)	0.37557 (16)	0.17935 (4)	0.0302 (3)	
O7	0.04963 (4)	−0.16000 (15)	0.44959 (4)	0.0258 (3)	
O8	0.11516 (4)	0.06677 (16)	0.48935 (3)	0.0255 (3)	
C23	0.16239 (6)	0.1905 (2)	0.25797 (5)	0.0188 (3)	
C24	0.11291 (6)	0.2176 (2)	0.24310 (5)	0.0185 (3)	
C25	0.07877 (6)	0.1781 (2)	0.27969 (5)	0.0185 (3)	
H25	0.0444	0.1382	0.2677	0.022*	
C25A	0.10949 (6)	0.0350 (2)	0.30487 (5)	0.0193 (3)	
C25B	0.10346 (6)	0.0281 (2)	0.35363 (5)	0.0192 (3)	
C26	0.07316 (6)	−0.0761 (2)	0.37696 (5)	0.0204 (3)	
H26	0.0508	−0.1554	0.3623	0.024*	
C27	0.07641 (6)	−0.0616 (2)	0.42257 (5)	0.0214 (4)	
C28	0.10869 (6)	0.0601 (2)	0.44392 (5)	0.0217 (4)	
C29	0.13786 (6)	0.1658 (2)	0.42003 (5)	0.0219 (4)	
H29	0.1591	0.2493	0.4343	0.026*	
C29A	0.13541 (6)	0.1474 (2)	0.37498 (5)	0.0205 (3)	
C30	0.16122 (6)	0.2481 (2)	0.34132 (5)	0.0204 (4)	
H30	0.1958	0.2889	0.3526	0.025*	
C30A	0.16310 (6)	0.1158 (2)	0.30369 (5)	0.0198 (3)	
H30A	0.1911	0.0318	0.3098	0.024*	
C31	0.12553 (6)	0.3929 (2)	0.32492 (5)	0.0210 (4)	
H31A	0.1217	0.4732	0.3491	0.025*	
H31B	0.1406	0.4545	0.3012	0.025*	
N32	0.07522 (5)	0.32672 (17)	0.30861 (4)	0.0199 (3)	
C33	0.21087 (6)	0.2266 (2)	0.23888 (5)	0.0201 (3)	
C34	0.21906 (6)	0.1952 (2)	0.19520 (5)	0.0259 (4)	
H34	0.1926	0.1476	0.1761	0.031*	
C35	0.26551 (7)	0.2328 (2)	0.17949 (6)	0.0310 (4)	
H35	0.2705	0.2098	0.1498	0.037*	
C36	0.30473 (7)	0.3035 (2)	0.20663 (6)	0.0293 (4)	
H36	0.3362	0.3308	0.1956	0.035*	
C37	0.29726 (6)	0.3338 (2)	0.25022 (6)	0.0279 (4)	
H37	0.3237	0.3829	0.2691	0.033*	
C38	0.25138 (6)	0.2928 (2)	0.26632 (5)	0.0235 (4)	
H38	0.2473	0.3097	0.2964	0.028*	
C39	0.09013 (6)	0.2923 (2)	0.20193 (5)	0.0209 (4)	
C40	0.10380 (8)	0.4429 (3)	0.13759 (6)	0.0380 (5)	
H40A	0.0890	0.3515	0.1191	0.057*	
H40B	0.1315	0.4960	0.1233	0.057*	
H40C	0.0776	0.5272	0.1420	0.057*	
C41	0.10082 (6)	−0.1333 (2)	0.28122 (5)	0.0230 (4)	
H41A	0.1229	−0.2193	0.2958	0.035*	
H41B	0.1088	−0.1213	0.2508	0.035*	
H41C	0.0652	−0.1672	0.2819	0.035*	
C42	0.07612 (7)	0.1543 (2)	0.50948 (5)	0.0289 (4)	
H42A	0.0696	0.2630	0.4949	0.043*	
H42B	0.0870	0.1734	0.5404	0.043*	
H42C	0.0449	0.0867	0.5070	0.043*	
C43	0.02597 (7)	−0.3088 (2)	0.43054 (6)	0.0338 (4)	
H43A	−0.0009	−0.2763	0.4080	0.051*	
H43B	0.0112	−0.3745	0.4533	0.051*	
H43C	0.0515	−0.3771	0.4173	0.051*	
C44	0.04325 (6)	0.4598 (2)	0.28805 (5)	0.0245 (4)	
H44A	0.0379	0.5475	0.3096	0.037*	
H44B	0.0104	0.4122	0.2769	0.037*	
H44C	0.0600	0.5089	0.2638	0.037*	
O9	0.38416 (4)	0.20757 (16)	0.51668 (4)	0.0281 (3)	
O10	0.46237 (4)	0.31084 (16)	0.50926 (4)	0.0283 (3)	
O11	0.36852 (5)	−0.25476 (16)	0.76825 (4)	0.0337 (3)	
O12	0.40905 (5)	−0.00499 (16)	0.81049 (4)	0.0346 (3)	
C45	0.49830 (6)	0.1339 (2)	0.59067 (5)	0.0194 (3)	
C46	0.44971 (6)	0.1511 (2)	0.57266 (5)	0.0191 (3)	
C47	0.41323 (6)	0.0987 (2)	0.60641 (5)	0.0204 (4)	
H47	0.3809	0.0510	0.5920	0.025*	
C47A	0.44527 (6)	−0.0399 (2)	0.63274 (5)	0.0208 (4)	
C47B	0.43428 (6)	−0.0539 (2)	0.68019 (5)	0.0217 (4)	
C48	0.40304 (6)	−0.1680 (2)	0.70002 (5)	0.0246 (4)	
H48	0.3862	−0.2562	0.6836	0.030*	
C49	0.39714 (6)	−0.1497 (2)	0.74438 (5)	0.0261 (4)	
C50	0.42073 (7)	−0.0153 (2)	0.76786 (5)	0.0274 (4)	
C51	0.45268 (6)	0.0966 (2)	0.74807 (5)	0.0263 (4)	
H51	0.4691	0.1863	0.7642	0.032*	
C51A	0.45971 (6)	0.0730 (2)	0.70407 (5)	0.0233 (4)	
C52	0.48757 (6)	0.1839 (2)	0.67344 (5)	0.0221 (4)	
H52	0.5199	0.2318	0.6878	0.027*	
C52A	0.49672 (6)	0.0566 (2)	0.63598 (5)	0.0206 (4)	
H52A	0.5263	−0.0194	0.6439	0.025*	
C53	0.45036 (6)	0.3205 (2)	0.65498 (5)	0.0223 (4)	
H53A	0.4424	0.3980	0.6787	0.027*	
H53B	0.4662	0.3871	0.6326	0.027*	
N54	0.40313 (5)	0.24188 (18)	0.63536 (4)	0.0219 (3)	
C55	0.54798 (6)	0.1758 (2)	0.57438 (5)	0.0201 (3)	
C56	0.55974 (6)	0.1444 (2)	0.53162 (5)	0.0235 (4)	
H56	0.5348	0.0956	0.5112	0.028*	
C57	0.60753 (6)	0.1839 (2)	0.51869 (6)	0.0273 (4)	
H57	0.6151	0.1607	0.4896	0.033*	
C58	0.64441 (6)	0.2571 (2)	0.54792 (5)	0.0258 (4)	
H58	0.6768	0.2855	0.5388	0.031*	
C59	0.63324 (6)	0.2882 (2)	0.59062 (5)	0.0253 (4)	
H59	0.6581	0.3390	0.6108	0.030*	
C60	0.58591 (6)	0.2452 (2)	0.60395 (5)	0.0229 (4)	
H60	0.5792	0.2630	0.6335	0.027*	
C61	0.42839 (6)	0.2241 (2)	0.53060 (5)	0.0212 (4)	
C62	0.44417 (7)	0.3744 (3)	0.46658 (5)	0.0337 (4)	
H62A	0.4133	0.4410	0.4689	0.050*	
H62B	0.4366	0.2796	0.4466	0.050*	
H62C	0.4704	0.4457	0.4553	0.050*	
C63	0.44378 (7)	−0.2058 (2)	0.60785 (5)	0.0260 (4)	
H63A	0.4673	−0.2860	0.6231	0.039*	
H63B	0.4538	−0.1864	0.5783	0.039*	
H63C	0.4092	−0.2519	0.6061	0.039*	
C64	0.42125 (8)	0.1456 (3)	0.83337 (6)	0.0406 (5)	
H64A	0.4076	0.1415	0.8620	0.061*	
H64B	0.4064	0.2415	0.8167	0.061*	
H64C	0.4583	0.1586	0.8374	0.061*	
C65	0.34943 (7)	−0.4060 (2)	0.74740 (6)	0.0362 (5)	
H65A	0.3315	−0.4725	0.7681	0.054*	
H65B	0.3778	−0.4719	0.7378	0.054*	
H65C	0.3259	−0.3770	0.7220	0.054*	
C66	0.36905 (6)	0.3679 (2)	0.61390 (5)	0.0253 (4)	
H66A	0.3386	0.3120	0.6001	0.038*	
H66B	0.3865	0.4270	0.5915	0.038*	
H66C	0.3592	0.4492	0.6356	0.038*	

### Atomic displacement parameters (Å^2^)

**Table:**

	*U*^11^	*U*^22^	*U*^33^	*U*^12^	*U*^13^	*U*^23^
O1	0.0238 (6)	0.0403 (8)	0.0228 (6)	0.0006 (6)	−0.0001 (5)	−0.0031 (5)
O2	0.0268 (6)	0.0402 (8)	0.0210 (6)	0.0067 (6)	0.0022 (5)	−0.0091 (5)
O3	0.0314 (7)	0.0264 (7)	0.0226 (6)	0.0071 (5)	0.0060 (5)	−0.0020 (5)
O4	0.0300 (6)	0.0262 (7)	0.0170 (5)	−0.0005 (5)	0.0025 (5)	0.0001 (5)
C1	0.0208 (8)	0.0184 (9)	0.0213 (8)	0.0004 (7)	0.0033 (6)	0.0016 (7)
C2	0.0204 (8)	0.0195 (9)	0.0196 (8)	−0.0001 (7)	0.0027 (6)	0.0007 (7)
C3	0.0189 (8)	0.0208 (9)	0.0186 (7)	0.0017 (7)	0.0013 (6)	0.0009 (7)
C3A	0.0195 (8)	0.0193 (9)	0.0198 (8)	0.0003 (7)	0.0024 (6)	−0.0008 (7)
C3B	0.0185 (8)	0.0196 (9)	0.0209 (8)	−0.0015 (7)	0.0012 (6)	−0.0021 (7)
C4	0.0198 (8)	0.0205 (9)	0.0224 (8)	−0.0007 (7)	0.0002 (6)	0.0004 (7)
C5	0.0191 (8)	0.0224 (9)	0.0216 (8)	−0.0004 (7)	0.0022 (6)	−0.0044 (7)
C6	0.0208 (8)	0.0242 (9)	0.0165 (7)	−0.0040 (7)	−0.0002 (6)	−0.0011 (7)
C7	0.0213 (8)	0.0218 (9)	0.0217 (8)	−0.0008 (7)	−0.0011 (6)	0.0003 (7)
C7A	0.0177 (8)	0.0193 (9)	0.0215 (8)	−0.0021 (7)	−0.0005 (6)	−0.0023 (7)
C8	0.0203 (8)	0.0228 (9)	0.0181 (8)	0.0033 (7)	0.0007 (6)	0.0005 (7)
C8A	0.0174 (8)	0.0209 (9)	0.0218 (8)	−0.0006 (7)	0.0014 (6)	−0.0018 (7)
C9	0.0211 (8)	0.0197 (9)	0.0214 (8)	0.0022 (7)	0.0028 (6)	0.0015 (7)
N10	0.0186 (7)	0.0199 (7)	0.0214 (7)	0.0002 (6)	0.0011 (5)	−0.0003 (6)
C11	0.0196 (8)	0.0214 (9)	0.0207 (8)	−0.0027 (7)	0.0025 (6)	−0.0013 (7)
C12	0.0264 (9)	0.0306 (10)	0.0308 (9)	0.0036 (8)	0.0061 (7)	0.0076 (8)
C13	0.0310 (10)	0.0354 (11)	0.0345 (10)	−0.0002 (8)	0.0146 (8)	0.0077 (9)
C14	0.0212 (9)	0.0313 (11)	0.0367 (10)	−0.0027 (8)	0.0085 (7)	−0.0046 (8)
C15	0.0205 (9)	0.0378 (11)	0.0271 (9)	0.0037 (8)	−0.0002 (7)	−0.0032 (8)
C16	0.0222 (8)	0.0329 (10)	0.0191 (8)	0.0027 (8)	0.0010 (6)	−0.0010 (7)
C17	0.0230 (9)	0.0224 (9)	0.0199 (8)	0.0005 (7)	0.0048 (6)	0.0016 (7)
C18	0.0402 (11)	0.0440 (12)	0.0203 (9)	0.0054 (9)	0.0020 (7)	−0.0089 (8)
C19	0.0274 (9)	0.0227 (10)	0.0227 (8)	−0.0006 (7)	0.0021 (7)	−0.0007 (7)
C20	0.0361 (10)	0.0325 (11)	0.0211 (8)	0.0020 (8)	0.0006 (7)	0.0061 (8)
C21	0.0351 (10)	0.0272 (10)	0.0308 (9)	0.0084 (8)	0.0067 (8)	−0.0024 (8)
C22	0.0208 (8)	0.0247 (10)	0.0260 (8)	−0.0038 (7)	0.0022 (7)	−0.0037 (7)
O5	0.0254 (6)	0.0397 (8)	0.0259 (6)	−0.0022 (6)	−0.0025 (5)	0.0053 (6)
O6	0.0285 (7)	0.0382 (8)	0.0235 (6)	−0.0069 (6)	0.0000 (5)	0.0125 (6)
O7	0.0278 (6)	0.0281 (7)	0.0222 (6)	−0.0059 (5)	0.0058 (5)	0.0051 (5)
O8	0.0254 (6)	0.0354 (7)	0.0157 (5)	0.0021 (5)	0.0023 (4)	0.0013 (5)
C23	0.0211 (8)	0.0175 (9)	0.0181 (7)	−0.0006 (7)	0.0031 (6)	−0.0023 (6)
C24	0.0211 (8)	0.0176 (9)	0.0171 (7)	−0.0017 (7)	0.0027 (6)	−0.0006 (6)
C25	0.0192 (8)	0.0201 (9)	0.0164 (7)	−0.0016 (7)	0.0027 (6)	−0.0004 (6)
C25A	0.0204 (8)	0.0205 (9)	0.0170 (7)	−0.0011 (7)	0.0011 (6)	0.0007 (7)
C25B	0.0173 (8)	0.0200 (9)	0.0203 (8)	0.0006 (7)	0.0008 (6)	0.0011 (7)
C26	0.0194 (8)	0.0201 (9)	0.0213 (8)	−0.0006 (7)	0.0000 (6)	0.0020 (7)
C27	0.0206 (8)	0.0229 (9)	0.0211 (8)	0.0005 (7)	0.0032 (6)	0.0046 (7)
C28	0.0226 (8)	0.0269 (10)	0.0153 (7)	0.0024 (7)	0.0000 (6)	0.0026 (7)
C29	0.0205 (8)	0.0243 (9)	0.0206 (8)	−0.0027 (7)	0.0001 (6)	0.0004 (7)
C29A	0.0194 (8)	0.0227 (9)	0.0191 (8)	−0.0009 (7)	0.0008 (6)	0.0021 (7)
C30	0.0193 (8)	0.0247 (9)	0.0172 (7)	−0.0050 (7)	0.0014 (6)	−0.0005 (7)
C30A	0.0184 (8)	0.0221 (9)	0.0189 (8)	0.0006 (7)	0.0017 (6)	0.0005 (7)
C31	0.0242 (8)	0.0200 (9)	0.0191 (8)	−0.0040 (7)	0.0045 (6)	−0.0021 (7)
N32	0.0214 (7)	0.0184 (7)	0.0200 (7)	−0.0005 (6)	0.0026 (5)	−0.0018 (6)
C33	0.0201 (8)	0.0184 (9)	0.0223 (8)	0.0013 (7)	0.0041 (6)	0.0008 (7)
C34	0.0258 (9)	0.0305 (10)	0.0218 (8)	−0.0028 (8)	0.0046 (7)	−0.0058 (7)
C35	0.0302 (10)	0.0395 (12)	0.0248 (9)	−0.0016 (9)	0.0109 (7)	−0.0027 (8)
C36	0.0232 (9)	0.0323 (11)	0.0336 (10)	0.0014 (8)	0.0102 (7)	0.0021 (8)
C37	0.0206 (9)	0.0320 (11)	0.0314 (9)	−0.0012 (8)	0.0038 (7)	−0.0004 (8)
C38	0.0220 (8)	0.0291 (10)	0.0196 (8)	0.0000 (7)	0.0029 (6)	−0.0003 (7)
C39	0.0223 (9)	0.0212 (9)	0.0196 (8)	−0.0020 (7)	0.0034 (6)	−0.0019 (7)
C40	0.0441 (12)	0.0455 (13)	0.0230 (9)	−0.0081 (10)	−0.0040 (8)	0.0134 (9)
C41	0.0272 (9)	0.0196 (9)	0.0218 (8)	−0.0012 (7)	−0.0005 (7)	−0.0006 (7)
C42	0.0282 (9)	0.0388 (11)	0.0202 (8)	0.0005 (8)	0.0043 (7)	−0.0016 (8)
C43	0.0391 (11)	0.0301 (11)	0.0335 (10)	−0.0092 (9)	0.0108 (8)	0.0021 (8)
C44	0.0253 (9)	0.0226 (9)	0.0264 (8)	0.0021 (7)	0.0068 (7)	0.0019 (7)
O9	0.0229 (6)	0.0366 (8)	0.0243 (6)	−0.0016 (5)	−0.0010 (5)	0.0026 (5)
O10	0.0267 (6)	0.0341 (7)	0.0238 (6)	−0.0040 (5)	0.0006 (5)	0.0099 (5)
O11	0.0405 (7)	0.0343 (8)	0.0281 (6)	−0.0021 (6)	0.0128 (5)	0.0076 (6)
O12	0.0493 (8)	0.0340 (8)	0.0212 (6)	−0.0002 (6)	0.0071 (5)	0.0008 (6)
C45	0.0222 (8)	0.0170 (9)	0.0194 (8)	−0.0006 (7)	0.0046 (6)	−0.0021 (7)
C46	0.0197 (8)	0.0188 (9)	0.0189 (8)	−0.0016 (7)	0.0029 (6)	−0.0015 (7)
C47	0.0184 (8)	0.0237 (9)	0.0193 (8)	−0.0013 (7)	0.0018 (6)	0.0008 (7)
C47A	0.0198 (8)	0.0215 (9)	0.0211 (8)	−0.0003 (7)	0.0021 (6)	0.0016 (7)
C47B	0.0193 (8)	0.0239 (9)	0.0222 (8)	0.0020 (7)	0.0030 (6)	0.0035 (7)
C48	0.0236 (9)	0.0256 (10)	0.0248 (8)	0.0019 (7)	0.0027 (7)	0.0046 (7)
C49	0.0271 (9)	0.0263 (10)	0.0256 (9)	0.0031 (8)	0.0063 (7)	0.0072 (8)
C50	0.0324 (10)	0.0320 (11)	0.0184 (8)	0.0078 (8)	0.0056 (7)	0.0069 (7)
C51	0.0293 (9)	0.0275 (10)	0.0218 (8)	0.0036 (8)	0.0008 (7)	0.0009 (7)
C51A	0.0227 (8)	0.0268 (10)	0.0203 (8)	0.0040 (7)	0.0011 (6)	0.0039 (7)
C52	0.0227 (8)	0.0256 (10)	0.0181 (8)	−0.0027 (7)	0.0012 (6)	−0.0017 (7)
C52A	0.0179 (8)	0.0216 (9)	0.0225 (8)	0.0013 (7)	0.0021 (6)	0.0016 (7)
C53	0.0246 (9)	0.0217 (9)	0.0207 (8)	−0.0016 (7)	0.0034 (6)	−0.0013 (7)
N54	0.0204 (7)	0.0236 (8)	0.0219 (7)	0.0017 (6)	0.0033 (5)	−0.0003 (6)
C55	0.0194 (8)	0.0185 (9)	0.0227 (8)	0.0018 (7)	0.0031 (6)	0.0004 (7)
C56	0.0220 (8)	0.0260 (10)	0.0232 (8)	−0.0024 (7)	0.0046 (6)	−0.0043 (7)
C57	0.0274 (9)	0.0304 (10)	0.0252 (9)	0.0001 (8)	0.0087 (7)	−0.0013 (8)
C58	0.0208 (8)	0.0274 (10)	0.0303 (9)	−0.0008 (7)	0.0074 (7)	0.0010 (8)
C59	0.0198 (8)	0.0287 (10)	0.0273 (9)	−0.0023 (7)	0.0009 (7)	0.0020 (8)
C60	0.0226 (8)	0.0253 (9)	0.0211 (8)	0.0010 (7)	0.0031 (6)	0.0004 (7)
C61	0.0232 (9)	0.0209 (9)	0.0199 (8)	−0.0001 (7)	0.0045 (6)	−0.0018 (7)
C62	0.0393 (11)	0.0379 (12)	0.0235 (9)	−0.0027 (9)	0.0012 (8)	0.0098 (8)
C63	0.0274 (9)	0.0228 (10)	0.0277 (9)	−0.0012 (7)	0.0018 (7)	0.0016 (7)
C64	0.0562 (13)	0.0413 (13)	0.0230 (9)	−0.0053 (10)	−0.0031 (9)	−0.0061 (9)
C65	0.0394 (11)	0.0330 (11)	0.0373 (11)	−0.0078 (9)	0.0088 (8)	0.0058 (9)
C66	0.0251 (9)	0.0247 (10)	0.0267 (9)	0.0044 (7)	0.0057 (7)	0.0026 (7)

### Geometric parameters (Å, º)

**Table:**

O1—C17	1.2105 (19)	C31—N32	1.474 (2)
O2—C17	1.3477 (19)	C31—H31A	0.9900
O2—C18	1.447 (2)	C31—H31B	0.9900
O3—C5	1.3737 (19)	N32—C44	1.459 (2)
O3—C21	1.435 (2)	C33—C34	1.400 (2)
O4—C6	1.3741 (18)	C33—C38	1.403 (2)
O4—C20	1.425 (2)	C34—C35	1.390 (2)
C1—C2	1.355 (2)	C34—H34	0.9500
C1—C11	1.485 (2)	C35—C36	1.389 (3)
C1—C8A	1.527 (2)	C35—H35	0.9500
C2—C17	1.485 (2)	C36—C37	1.392 (2)
C2—C3	1.532 (2)	C36—H36	0.9500
C3—N10	1.485 (2)	C37—C38	1.387 (2)
C3—C3A	1.565 (2)	C37—H37	0.9500
C3—H3	1.0000	C38—H38	0.9500
C3A—C3B	1.516 (2)	C40—H40A	0.9800
C3A—C19	1.525 (2)	C40—H40B	0.9800
C3A—C8A	1.555 (2)	C40—H40C	0.9800
C3B—C7A	1.391 (2)	C41—H41A	0.9800
C3B—C4	1.393 (2)	C41—H41B	0.9800
C4—C5	1.396 (2)	C41—H41C	0.9800
C4—H4	0.9500	C42—H42A	0.9800
C5—C6	1.415 (2)	C42—H42B	0.9800
C6—C7	1.396 (2)	C42—H42C	0.9800
C7—C7A	1.394 (2)	C43—H43A	0.9800
C7—H7	0.9500	C43—H43B	0.9800
C7A—C8	1.524 (2)	C43—H43C	0.9800
C8—C9	1.541 (2)	C44—H44A	0.9800
C8—C8A	1.563 (2)	C44—H44B	0.9800
C8—H8	1.0000	C44—H44C	0.9800
C8A—H8A	1.0000	O9—C61	1.2148 (19)
C9—N10	1.475 (2)	O10—C61	1.346 (2)
C9—H9A	0.9900	O10—C62	1.446 (2)
C9—H9B	0.9900	O11—C49	1.379 (2)
N10—C22	1.458 (2)	O11—C65	1.430 (2)
C11—C12	1.393 (2)	O12—C50	1.3744 (19)
C11—C16	1.401 (2)	O12—C64	1.409 (2)
C12—C13	1.388 (2)	C45—C46	1.358 (2)
C12—H12	0.9500	C45—C55	1.483 (2)
C13—C14	1.389 (3)	C45—C52A	1.525 (2)
C13—H13	0.9500	C46—C61	1.481 (2)
C14—C15	1.387 (2)	C46—C47	1.534 (2)
C14—H14	0.9500	C47—N54	1.481 (2)
C15—C16	1.392 (2)	C47—C47A	1.567 (2)
C15—H15	0.9500	C47—H47	1.0000
C16—H16	0.9500	C47A—C47B	1.515 (2)
C18—H18A	0.9800	C47A—C63	1.520 (2)
C18—H18B	0.9800	C47A—C52A	1.555 (2)
C18—H18C	0.9800	C47B—C51A	1.383 (2)
C19—H19A	0.9800	C47B—C48	1.400 (2)
C19—H19B	0.9800	C48—C49	1.393 (2)
C19—H19C	0.9800	C48—H48	0.9500
C20—H20A	0.9800	C49—C50	1.402 (3)
C20—H20B	0.9800	C50—C51	1.400 (3)
C20—H20C	0.9800	C51—C51A	1.393 (2)
C21—H21A	0.9800	C51—H51	0.9500
C21—H21B	0.9800	C51A—C52	1.524 (2)
C21—H21C	0.9800	C52—C53	1.537 (2)
C22—H22A	0.9800	C52—C52A	1.566 (2)
C22—H22B	0.9800	C52—H52	1.0000
C22—H22C	0.9800	C52A—H52A	1.0000
O5—C39	1.2177 (19)	C53—N54	1.474 (2)
O6—C39	1.3414 (19)	C53—H53A	0.9900
O6—C40	1.443 (2)	C53—H53B	0.9900
O7—C27	1.3789 (19)	N54—C66	1.462 (2)
O7—C43	1.435 (2)	C55—C56	1.399 (2)
O8—C28	1.3919 (18)	C55—C60	1.403 (2)
O8—C42	1.428 (2)	C56—C57	1.392 (2)
C23—C24	1.362 (2)	C56—H56	0.9500
C23—C33	1.483 (2)	C57—C58	1.391 (2)
C23—C30A	1.523 (2)	C57—H57	0.9500
C24—C39	1.476 (2)	C58—C59	1.392 (2)
C24—C25	1.534 (2)	C58—H58	0.9500
C25—N32	1.484 (2)	C59—C60	1.392 (2)
C25—C25A	1.560 (2)	C59—H59	0.9500
C25—H25	1.0000	C60—H60	0.9500
C25A—C25B	1.521 (2)	C62—H62A	0.9800
C25A—C41	1.527 (2)	C62—H62B	0.9800
C25A—C30A	1.557 (2)	C62—H62C	0.9800
C25B—C26	1.392 (2)	C63—H63A	0.9800
C25B—C29A	1.393 (2)	C63—H63B	0.9800
C26—C27	1.401 (2)	C63—H63C	0.9800
C26—H26	0.9500	C64—H64A	0.9800
C27—C28	1.410 (2)	C64—H64B	0.9800
C28—C29	1.391 (2)	C64—H64C	0.9800
C29—C29A	1.387 (2)	C65—H65A	0.9800
C29—H29	0.9500	C65—H65B	0.9800
C29A—C30	1.515 (2)	C65—H65C	0.9800
C30—C31	1.541 (2)	C66—H66A	0.9800
C30—C30A	1.566 (2)	C66—H66B	0.9800
C30—H30	1.0000	C66—H66C	0.9800
C30A—H30A	1.0000		
			
C17—O2—C18	115.32 (13)	H31A—C31—H31B	108.1
C5—O3—C21	116.44 (13)	C44—N32—C31	111.03 (13)
C6—O4—C20	117.24 (13)	C44—N32—C25	112.34 (12)
C2—C1—C11	131.73 (15)	C31—N32—C25	112.55 (12)
C2—C1—C8A	108.29 (13)	C34—C33—C38	117.90 (15)
C11—C1—C8A	119.97 (13)	C34—C33—C23	123.82 (15)
C1—C2—C17	131.57 (15)	C38—C33—C23	118.26 (14)
C1—C2—C3	109.17 (13)	C35—C34—C33	120.70 (16)
C17—C2—C3	118.94 (13)	C35—C34—H34	119.6
N10—C3—C2	111.07 (13)	C33—C34—H34	119.6
N10—C3—C3A	109.91 (12)	C36—C35—C34	120.82 (16)
C2—C3—C3A	101.02 (12)	C36—C35—H35	119.6
N10—C3—H3	111.5	C34—C35—H35	119.6
C2—C3—H3	111.5	C35—C36—C37	119.05 (16)
C3A—C3—H3	111.5	C35—C36—H36	120.5
C3B—C3A—C19	114.39 (13)	C37—C36—H36	120.5
C3B—C3A—C8A	102.28 (12)	C38—C37—C36	120.35 (16)
C19—C3A—C8A	115.55 (13)	C38—C37—H37	119.8
C3B—C3A—C3	114.21 (13)	C36—C37—H37	119.8
C19—C3A—C3	111.78 (13)	C37—C38—C33	121.11 (15)
C8A—C3A—C3	97.13 (12)	C37—C38—H38	119.4
C7A—C3B—C4	121.05 (14)	C33—C38—H38	119.4
C7A—C3B—C3A	109.34 (14)	O5—C39—O6	122.77 (15)
C4—C3B—C3A	129.61 (15)	O5—C39—C24	123.36 (15)
C3B—C4—C5	118.62 (15)	O6—C39—C24	113.86 (13)
C3B—C4—H4	120.7	O6—C40—H40A	109.5
C5—C4—H4	120.7	O6—C40—H40B	109.5
O3—C5—C4	124.74 (15)	H40A—C40—H40B	109.5
O3—C5—C6	115.02 (14)	O6—C40—H40C	109.5
C4—C5—C6	120.24 (15)	H40A—C40—H40C	109.5
O4—C6—C7	125.03 (15)	H40B—C40—H40C	109.5
O4—C6—C5	114.44 (14)	C25A—C41—H41A	109.5
C7—C6—C5	120.52 (14)	C25A—C41—H41B	109.5
C7A—C7—C6	118.45 (15)	H41A—C41—H41B	109.5
C7A—C7—H7	120.8	C25A—C41—H41C	109.5
C6—C7—H7	120.8	H41A—C41—H41C	109.5
C3B—C7A—C7	121.01 (15)	H41B—C41—H41C	109.5
C3B—C7A—C8	109.04 (13)	O8—C42—H42A	109.5
C7—C7A—C8	129.64 (15)	O8—C42—H42B	109.5
C7A—C8—C9	107.86 (13)	H42A—C42—H42B	109.5
C7A—C8—C8A	101.22 (13)	O8—C42—H42C	109.5
C9—C8—C8A	108.69 (12)	H42A—C42—H42C	109.5
C7A—C8—H8	112.8	H42B—C42—H42C	109.5
C9—C8—H8	112.8	O7—C43—H43A	109.5
C8A—C8—H8	112.8	O7—C43—H43B	109.5
C1—C8A—C3A	102.94 (12)	H43A—C43—H43B	109.5
C1—C8A—C8	115.81 (14)	O7—C43—H43C	109.5
C3A—C8A—C8	100.12 (12)	H43A—C43—H43C	109.5
C1—C8A—H8A	112.3	H43B—C43—H43C	109.5
C3A—C8A—H8A	112.3	N32—C44—H44A	109.5
C8—C8A—H8A	112.3	N32—C44—H44B	109.5
N10—C9—C8	110.22 (13)	H44A—C44—H44B	109.5
N10—C9—H9A	109.6	N32—C44—H44C	109.5
C8—C9—H9A	109.6	H44A—C44—H44C	109.5
N10—C9—H9B	109.6	H44B—C44—H44C	109.5
C8—C9—H9B	109.6	C61—O10—C62	115.94 (13)
H9A—C9—H9B	108.1	C49—O11—C65	117.15 (14)
C22—N10—C9	110.70 (13)	C50—O12—C64	117.71 (15)
C22—N10—C3	113.17 (12)	C46—C45—C55	132.22 (15)
C9—N10—C3	112.43 (12)	C46—C45—C52A	108.11 (13)
C12—C11—C16	118.24 (15)	C55—C45—C52A	119.67 (14)
C12—C11—C1	122.36 (15)	C45—C46—C61	131.83 (14)
C16—C11—C1	119.36 (14)	C45—C46—C47	108.93 (13)
C13—C12—C11	121.27 (17)	C61—C46—C47	118.88 (13)
C13—C12—H12	119.4	N54—C47—C46	110.80 (13)
C11—C12—H12	119.4	N54—C47—C47A	110.20 (12)
C12—C13—C14	120.25 (16)	C46—C47—C47A	101.34 (12)
C12—C13—H13	119.9	N54—C47—H47	111.4
C14—C13—H13	119.9	C46—C47—H47	111.4
C15—C14—C13	119.00 (16)	C47A—C47—H47	111.4
C15—C14—H14	120.5	C47B—C47A—C63	114.94 (14)
C13—C14—H14	120.5	C47B—C47A—C52A	102.44 (13)
C14—C15—C16	120.99 (17)	C63—C47A—C52A	116.19 (14)
C14—C15—H15	119.5	C47B—C47A—C47	114.04 (13)
C16—C15—H15	119.5	C63—C47A—C47	111.00 (13)
C15—C16—C11	120.19 (15)	C52A—C47A—C47	96.70 (13)
C15—C16—H16	119.9	C51A—C47B—C48	121.09 (15)
C11—C16—H16	119.9	C51A—C47B—C47A	109.35 (14)
O1—C17—O2	122.95 (15)	C48—C47B—C47A	129.54 (15)
O1—C17—C2	122.93 (14)	C49—C48—C47B	118.67 (16)
O2—C17—C2	114.11 (14)	C49—C48—H48	120.7
O2—C18—H18A	109.5	C47B—C48—H48	120.7
O2—C18—H18B	109.5	O11—C49—C48	124.86 (16)
H18A—C18—H18B	109.5	O11—C49—C50	115.17 (14)
O2—C18—H18C	109.5	C48—C49—C50	119.96 (16)
H18A—C18—H18C	109.5	O12—C50—C51	124.83 (17)
H18B—C18—H18C	109.5	O12—C50—C49	114.11 (15)
C3A—C19—H19A	109.5	C51—C50—C49	121.06 (15)
C3A—C19—H19B	109.5	C51A—C51—C50	118.25 (17)
H19A—C19—H19B	109.5	C51A—C51—H51	120.9
C3A—C19—H19C	109.5	C50—C51—H51	120.9
H19A—C19—H19C	109.5	C47B—C51A—C51	120.81 (16)
H19B—C19—H19C	109.5	C47B—C51A—C52	109.30 (14)
O4—C20—H20A	109.5	C51—C51A—C52	129.37 (16)
O4—C20—H20B	109.5	C51A—C52—C53	107.87 (13)
H20A—C20—H20B	109.5	C51A—C52—C52A	101.29 (13)
O4—C20—H20C	109.5	C53—C52—C52A	108.58 (13)
H20A—C20—H20C	109.5	C51A—C52—H52	112.8
H20B—C20—H20C	109.5	C53—C52—H52	112.8
O3—C21—H21A	109.5	C52A—C52—H52	112.8
O3—C21—H21B	109.5	C45—C52A—C47A	103.53 (12)
H21A—C21—H21B	109.5	C45—C52A—C52	115.48 (14)
O3—C21—H21C	109.5	C47A—C52A—C52	99.98 (12)
H21A—C21—H21C	109.5	C45—C52A—H52A	112.3
H21B—C21—H21C	109.5	C47A—C52A—H52A	112.3
N10—C22—H22A	109.5	C52—C52A—H52A	112.3
N10—C22—H22B	109.5	N54—C53—C52	110.03 (14)
H22A—C22—H22B	109.5	N54—C53—H53A	109.7
N10—C22—H22C	109.5	C52—C53—H53A	109.7
H22A—C22—H22C	109.5	N54—C53—H53B	109.7
H22B—C22—H22C	109.5	C52—C53—H53B	109.7
C39—O6—C40	116.17 (13)	H53A—C53—H53B	108.2
C27—O7—C43	116.59 (13)	C66—N54—C53	111.07 (13)
C28—O8—C42	115.06 (12)	C66—N54—C47	112.94 (13)
C24—C23—C33	132.29 (14)	C53—N54—C47	112.27 (12)
C24—C23—C30A	107.67 (13)	C56—C55—C60	118.20 (15)
C33—C23—C30A	119.97 (13)	C56—C55—C45	123.61 (15)
C23—C24—C39	131.03 (15)	C60—C55—C45	118.14 (14)
C23—C24—C25	109.29 (13)	C57—C56—C55	120.68 (16)
C39—C24—C25	119.26 (13)	C57—C56—H56	119.7
N32—C25—C24	110.41 (13)	C55—C56—H56	119.7
N32—C25—C25A	109.79 (12)	C58—C57—C56	120.66 (15)
C24—C25—C25A	101.45 (12)	C58—C57—H57	119.7
N32—C25—H25	111.6	C56—C57—H57	119.7
C24—C25—H25	111.6	C57—C58—C59	119.18 (15)
C25A—C25—H25	111.6	C57—C58—H58	120.4
C25B—C25A—C41	114.38 (13)	C59—C58—H58	120.4
C25B—C25A—C30A	102.27 (12)	C60—C59—C58	120.32 (16)
C41—C25A—C30A	116.53 (13)	C60—C59—H59	119.8
C25B—C25A—C25	114.79 (13)	C58—C59—H59	119.8
C41—C25A—C25	110.51 (12)	C59—C60—C55	120.90 (15)
C30A—C25A—C25	97.07 (12)	C59—C60—H60	119.5
C26—C25B—C29A	120.83 (14)	C55—C60—H60	119.5
C26—C25B—C25A	130.04 (14)	O9—C61—O10	123.04 (15)
C29A—C25B—C25A	109.12 (13)	O9—C61—C46	122.97 (15)
C25B—C26—C27	118.64 (15)	O10—C61—C46	113.99 (14)
C25B—C26—H26	120.7	O10—C62—H62A	109.5
C27—C26—H26	120.7	O10—C62—H62B	109.5
O7—C27—C26	124.50 (15)	H62A—C62—H62B	109.5
O7—C27—C28	115.37 (14)	O10—C62—H62C	109.5
C26—C27—C28	120.13 (14)	H62A—C62—H62C	109.5
C29—C28—O8	118.79 (14)	H62B—C62—H62C	109.5
C29—C28—C27	120.49 (14)	C47A—C63—H63A	109.5
O8—C28—C27	120.50 (14)	C47A—C63—H63B	109.5
C29A—C29—C28	118.94 (15)	H63A—C63—H63B	109.5
C29A—C29—H29	120.5	C47A—C63—H63C	109.5
C28—C29—H29	120.5	H63A—C63—H63C	109.5
C29—C29A—C25B	120.93 (15)	H63B—C63—H63C	109.5
C29—C29A—C30	129.89 (15)	O12—C64—H64A	109.5
C25B—C29A—C30	109.08 (13)	O12—C64—H64B	109.5
C29A—C30—C31	108.47 (13)	H64A—C64—H64B	109.5
C29A—C30—C30A	101.31 (13)	O12—C64—H64C	109.5
C31—C30—C30A	108.45 (12)	H64A—C64—H64C	109.5
C29A—C30—H30	112.6	H64B—C64—H64C	109.5
C31—C30—H30	112.6	O11—C65—H65A	109.5
C30A—C30—H30	112.6	O11—C65—H65B	109.5
C23—C30A—C25A	104.16 (12)	H65A—C65—H65B	109.5
C23—C30A—C30	114.93 (13)	O11—C65—H65C	109.5
C25A—C30A—C30	99.80 (12)	H65A—C65—H65C	109.5
C23—C30A—H30A	112.3	H65B—C65—H65C	109.5
C25A—C30A—H30A	112.3	N54—C66—H66A	109.5
C30—C30A—H30A	112.3	N54—C66—H66B	109.5
N32—C31—C30	110.63 (13)	H66A—C66—H66B	109.5
N32—C31—H31A	109.5	N54—C66—H66C	109.5
C30—C31—H31A	109.5	H66A—C66—H66C	109.5
N32—C31—H31B	109.5	H66B—C66—H66C	109.5
C30—C31—H31B	109.5		
			
C11—C1—C2—C17	−4.3 (3)	C29—C29A—C30—C30A	154.58 (17)
C8A—C1—C2—C17	177.21 (17)	C25B—C29A—C30—C30A	−29.07 (16)
C11—C1—C2—C3	−177.48 (16)	C24—C23—C30A—C25A	23.32 (17)
C8A—C1—C2—C3	3.99 (18)	C33—C23—C30A—C25A	−159.21 (14)
C1—C2—C3—N10	85.35 (16)	C24—C23—C30A—C30	−84.78 (16)
C17—C2—C3—N10	−88.86 (17)	C33—C23—C30A—C30	92.68 (17)
C1—C2—C3—C3A	−31.19 (17)	C25B—C25A—C30A—C23	−157.64 (12)
C17—C2—C3—C3A	154.60 (14)	C41—C25A—C30A—C23	76.86 (16)
N10—C3—C3A—C3B	32.76 (18)	C25—C25A—C30A—C23	−40.30 (14)
C2—C3—C3A—C3B	150.15 (13)	C25B—C25A—C30A—C30	−38.65 (15)
N10—C3—C3A—C19	164.63 (13)	C41—C25A—C30A—C30	−164.14 (13)
C2—C3—C3A—C19	−77.98 (15)	C25—C25A—C30A—C30	78.70 (13)
N10—C3—C3A—C8A	−74.17 (14)	C29A—C30—C30A—C23	151.59 (13)
C2—C3—C3A—C8A	43.22 (14)	C31—C30—C30A—C23	37.56 (17)
C19—C3A—C3B—C7A	149.06 (14)	C29A—C30—C30A—C25A	40.86 (14)
C8A—C3A—C3B—C7A	23.36 (17)	C31—C30—C30A—C25A	−73.17 (14)
C3—C3A—C3B—C7A	−80.35 (16)	C29A—C30—C31—N32	−54.65 (16)
C19—C3A—C3B—C4	−30.9 (2)	C30A—C30—C31—N32	54.58 (16)
C8A—C3A—C3B—C4	−156.63 (16)	C30—C31—N32—C44	−172.92 (12)
C3—C3A—C3B—C4	99.7 (2)	C30—C31—N32—C25	−46.02 (16)
C7A—C3B—C4—C5	0.4 (2)	C24—C25—N32—C44	73.76 (16)
C3A—C3B—C4—C5	−179.63 (15)	C25A—C25—N32—C44	−175.20 (12)
C21—O3—C5—C4	10.8 (2)	C24—C25—N32—C31	−52.44 (16)
C21—O3—C5—C6	−169.50 (14)	C25A—C25—N32—C31	58.60 (16)
C3B—C4—C5—O3	−177.74 (15)	C24—C23—C33—C34	−43.9 (3)
C3B—C4—C5—C6	2.6 (2)	C30A—C23—C33—C34	139.35 (17)
C20—O4—C6—C7	12.3 (2)	C24—C23—C33—C38	137.56 (19)
C20—O4—C6—C5	−168.59 (14)	C30A—C23—C33—C38	−39.2 (2)
O3—C5—C6—O4	−2.0 (2)	C38—C33—C34—C35	−1.7 (3)
C4—C5—C6—O4	177.74 (14)	C23—C33—C34—C35	179.80 (17)
O3—C5—C6—C7	177.21 (14)	C33—C34—C35—C36	−0.5 (3)
C4—C5—C6—C7	−3.1 (2)	C34—C35—C36—C37	1.1 (3)
O4—C6—C7—C7A	179.67 (14)	C35—C36—C37—C38	0.4 (3)
C5—C6—C7—C7A	0.6 (2)	C36—C37—C38—C33	−2.7 (3)
C4—C3B—C7A—C7	−2.9 (2)	C34—C33—C38—C37	3.2 (3)
C3A—C3B—C7A—C7	177.10 (14)	C23—C33—C38—C37	−178.16 (16)
C4—C3B—C7A—C8	−177.06 (14)	C40—O6—C39—O5	−3.2 (2)
C3A—C3B—C7A—C8	2.94 (18)	C40—O6—C39—C24	176.56 (15)
C6—C7—C7A—C3B	2.4 (2)	C23—C24—C39—O5	165.36 (17)
C6—C7—C7A—C8	175.21 (15)	C25—C24—C39—O5	−22.9 (2)
C3B—C7A—C8—C9	86.25 (16)	C23—C24—C39—O6	−14.4 (3)
C7—C7A—C8—C9	−87.25 (19)	C25—C24—C39—O6	157.40 (14)
C3B—C7A—C8—C8A	−27.78 (16)	C55—C45—C46—C61	−2.5 (3)
C7—C7A—C8—C8A	158.72 (16)	C52A—C45—C46—C61	177.20 (16)
C2—C1—C8A—C3A	25.11 (17)	C55—C45—C46—C47	−175.48 (16)
C11—C1—C8A—C3A	−153.63 (14)	C52A—C45—C46—C47	4.22 (18)
C2—C1—C8A—C8	−83.04 (17)	C45—C46—C47—N54	85.56 (16)
C11—C1—C8A—C8	98.23 (17)	C61—C46—C47—N54	−88.48 (17)
C3B—C3A—C8A—C1	−158.40 (13)	C45—C46—C47—C47A	−31.37 (17)
C19—C3A—C8A—C1	76.66 (16)	C61—C46—C47—C47A	154.60 (14)
C3—C3A—C8A—C1	−41.65 (14)	N54—C47—C47A—C47B	32.61 (18)
C3B—C3A—C8A—C8	−38.74 (14)	C46—C47—C47A—C47B	149.97 (13)
C19—C3A—C8A—C8	−163.67 (13)	N54—C47—C47A—C63	164.35 (13)
C3—C3A—C8A—C8	78.01 (13)	C46—C47—C47A—C63	−78.29 (15)
C7A—C8—C8A—C1	149.93 (13)	N54—C47—C47A—C52A	−74.25 (14)
C9—C8—C8A—C1	36.53 (18)	C46—C47—C47A—C52A	43.12 (14)
C7A—C8—C8A—C3A	40.12 (14)	C63—C47A—C47B—C51A	150.67 (14)
C9—C8—C8A—C3A	−73.29 (15)	C52A—C47A—C47B—C51A	23.74 (17)
C7A—C8—C9—N10	−53.83 (16)	C47—C47A—C47B—C51A	−79.52 (17)
C8A—C8—C9—N10	55.13 (16)	C63—C47A—C47B—C48	−31.1 (2)
C8—C9—N10—C22	−174.44 (12)	C52A—C47A—C47B—C48	−158.03 (17)
C8—C9—N10—C3	−46.77 (16)	C47—C47A—C47B—C48	98.7 (2)
C2—C3—N10—C22	74.47 (16)	C51A—C47B—C48—C49	1.2 (2)
C3A—C3—N10—C22	−174.60 (12)	C47A—C47B—C48—C49	−176.84 (16)
C2—C3—N10—C9	−51.88 (17)	C65—O11—C49—C48	9.1 (2)
C3A—C3—N10—C9	59.06 (16)	C65—O11—C49—C50	−171.75 (15)
C2—C1—C11—C12	−48.2 (3)	C47B—C48—C49—O11	−178.54 (15)
C8A—C1—C11—C12	130.16 (17)	C47B—C48—C49—C50	2.4 (2)
C2—C1—C11—C16	134.47 (19)	C64—O12—C50—C51	13.6 (3)
C8A—C1—C11—C16	−47.1 (2)	C64—O12—C50—C49	−165.73 (16)
C16—C11—C12—C13	−1.0 (3)	O11—C49—C50—O12	−3.4 (2)
C1—C11—C12—C13	−178.31 (17)	C48—C49—C50—O12	175.73 (15)
C11—C12—C13—C14	−0.9 (3)	O11—C49—C50—C51	177.20 (15)
C12—C13—C14—C15	1.3 (3)	C48—C49—C50—C51	−3.6 (3)
C13—C14—C15—C16	0.2 (3)	O12—C50—C51—C51A	−178.05 (15)
C14—C15—C16—C11	−2.2 (3)	C49—C50—C51—C51A	1.2 (3)
C12—C11—C16—C15	2.5 (3)	C48—C47B—C51A—C51	−3.6 (3)
C1—C11—C16—C15	179.93 (16)	C47A—C47B—C51A—C51	174.76 (15)
C18—O2—C17—O1	−2.2 (2)	C48—C47B—C51A—C52	−176.13 (15)
C18—O2—C17—C2	177.75 (15)	C47A—C47B—C51A—C52	2.28 (18)
C1—C2—C17—O1	172.56 (18)	C50—C51—C51A—C47B	2.4 (2)
C3—C2—C17—O1	−14.8 (2)	C50—C51—C51A—C52	173.18 (16)
C1—C2—C17—O2	−7.4 (3)	C47B—C51A—C52—C53	86.89 (16)
C3—C2—C17—O2	165.25 (14)	C51—C51A—C52—C53	−84.8 (2)
C33—C23—C24—C39	0.6 (3)	C47B—C51A—C52—C52A	−27.06 (17)
C30A—C23—C24—C39	177.66 (16)	C51—C51A—C52—C52A	161.30 (17)
C33—C23—C24—C25	−171.75 (16)	C46—C45—C52A—C47A	24.97 (17)
C30A—C23—C24—C25	5.29 (18)	C55—C45—C52A—C47A	−155.29 (14)
C23—C24—C25—N32	84.75 (16)	C46—C45—C52A—C52	−83.22 (17)
C39—C24—C25—N32	−88.66 (17)	C55—C45—C52A—C52	96.52 (17)
C23—C24—C25—C25A	−31.61 (16)	C47B—C47A—C52A—C45	−157.99 (13)
C39—C24—C25—C25A	154.98 (14)	C63—C47A—C52A—C45	75.88 (17)
N32—C25—C25A—C25B	32.49 (18)	C47—C47A—C52A—C45	−41.51 (14)
C24—C25—C25A—C25B	149.30 (13)	C47B—C47A—C52A—C52	−38.55 (15)
N32—C25—C25A—C41	163.65 (12)	C63—C47A—C52A—C52	−164.68 (13)
C24—C25—C25A—C41	−79.54 (15)	C47—C47A—C52A—C52	77.94 (13)
N32—C25—C25A—C30A	−74.56 (14)	C51A—C52—C52A—C45	149.79 (13)
C24—C25—C25A—C30A	42.26 (13)	C53—C52—C52A—C45	36.38 (18)
C41—C25A—C25B—C26	−29.3 (2)	C51A—C52—C52A—C47A	39.48 (15)
C30A—C25A—C25B—C26	−156.20 (16)	C53—C52—C52A—C47A	−73.93 (15)
C25—C25A—C25B—C26	100.0 (2)	C51A—C52—C53—N54	−53.32 (16)
C41—C25A—C25B—C29A	149.53 (14)	C52A—C52—C53—N54	55.67 (16)
C30A—C25A—C25B—C29A	22.64 (17)	C52—C53—N54—C66	−174.61 (12)
C25—C25A—C25B—C29A	−81.20 (17)	C52—C53—N54—C47	−47.09 (17)
C29A—C25B—C26—C27	−1.6 (2)	C46—C47—N54—C66	74.48 (16)
C25A—C25B—C26—C27	177.10 (15)	C47A—C47—N54—C66	−174.19 (13)
C43—O7—C27—C26	14.1 (2)	C46—C47—N54—C53	−52.04 (17)
C43—O7—C27—C28	−165.90 (15)	C47A—C47—N54—C53	59.29 (16)
C25B—C26—C27—O7	−178.15 (15)	C46—C45—C55—C56	−42.3 (3)
C25B—C26—C27—C28	1.9 (2)	C52A—C45—C55—C56	138.03 (17)
C42—O8—C28—C29	103.54 (18)	C46—C45—C55—C60	140.13 (19)
C42—O8—C28—C27	−81.8 (2)	C52A—C45—C55—C60	−39.5 (2)
O7—C27—C28—C29	179.56 (15)	C60—C55—C56—C57	−1.1 (3)
C26—C27—C28—C29	−0.5 (2)	C45—C55—C56—C57	−178.70 (16)
O7—C27—C28—O8	5.0 (2)	C55—C56—C57—C58	−0.7 (3)
C26—C27—C28—O8	−175.00 (14)	C56—C57—C58—C59	1.0 (3)
O8—C28—C29—C29A	173.37 (14)	C57—C58—C59—C60	0.5 (3)
C27—C28—C29—C29A	−1.2 (2)	C58—C59—C60—C55	−2.4 (3)
C28—C29—C29A—C25B	1.5 (2)	C56—C55—C60—C59	2.7 (2)
C28—C29—C29A—C30	177.52 (16)	C45—C55—C60—C59	−179.63 (15)
C26—C25B—C29A—C29	−0.1 (2)	C62—O10—C61—O9	−4.4 (2)
C25A—C25B—C29A—C29	−179.06 (15)	C62—O10—C61—C46	175.83 (14)
C26—C25B—C29A—C30	−176.84 (14)	C45—C46—C61—O9	168.95 (17)
C25A—C25B—C29A—C30	4.20 (18)	C47—C46—C61—O9	−18.6 (2)
C29—C29A—C30—C31	−91.4 (2)	C45—C46—C61—O10	−11.3 (3)
C25B—C29A—C30—C31	84.96 (16)	C47—C46—C61—O10	161.13 (14)

## References

[bb1] Battye, T. G. G., Kontogiannis, L., Johnson, O., Powell, H. R. & Leslie, A. G. W. (2011). *Acta Cryst.* D**67**, 271–281. 10.1107/S0907444910048675 PMC3069742 21460445

[bb2] Batzold, F. H., Covey, D. F. & Robinson, C. H. (1977). *Cancer Treat. Rep.* **61**, 255–257. 872131

[bb3] Batzold, F. H. & Robinson, C. H. (1975). *J. Am. Chem. Soc.* **97**, 2576–2578. 10.1021/ja00842a064 1133425

[bb4] Batzold, F. H. & Robinson, C. H. (1976). *J. Org. Chem.* **41**, 313–317. 10.1021/jo00864a028 1245907

[bb5] Bohlmann, F., Gupta, R. K., Jakupovic, J., King, R. M. & Robinson, H. (1980). *Liebigs Ann. Chem.* pp. 1904–1906.

[bb6] Doyle, R. A. (2011). *Marccd software manual*. Rayonix L. L. C., Evanston, IL 60201, USA.

[bb7] Evans, P. (2006). *Acta Cryst.* D**62**, 72–82. 10.1107/S0907444905036693 16369096

[bb8] Jakupovic, J., Schmeda-Hirschmann, G., Schuster, A., Zdero, C., Bohlmann, F., King, R. M., Robinson, H. & Pickardt, J. (1985). *Phytochemistry*, **25**, 145–158.

[bb9] Krause, N. & Hoffmann-Röder, A. (2004). *Modern Allene Chemistry*, Vol. 2, edited by N. Krause & A. S. K. Hashmi, pp. 997–1032. Weinheim: Wiley-VCH Verlag GmbH & Co.

[bb10] Perscheid, M., Schollmeyer, D. & Nubbemeyer, U. (2011). *Eur. J. Org. Chem.* pp. 5250–5253.

[bb11] Sashida, H. & Tsuchiya, T. (1984). *Chem. Pharm. Bull.* **32**, 4600–4607.

[bb16] Sheldrick, G. M. (2008). *Acta Cryst* A**64**, 112–122. 10.1107/S0108767307043930 18156677

[bb12] Sheldrick, G. M. (2015*a*). *Acta Cryst.* A**71**, 3–8.

[bb13] Sheldrick, G. M. (2015*b*). *Acta Cryst.* C**71**, 3–8.

[bb14] Voskressensky, L. G., Titov, A. A., Dzhankaziev, M. S., Borisova, T. N., Kobzev, M. S., Dorovatovskii, P. V., Khrustalev, V. N., Aksenov, A. V. & Varlamov, A. V. (2017). *New J. Chem.* **41**, 1902–1904.

[bb15] Warning, U., Jakupovic, J., Bohlmann, F. & Jones, S. B. (1987). *Liebigs Ann. Chem.* pp. 467–468.

